# Insights into the oral health beliefs and practices of mothers from a north London Orthodox Jewish community

**DOI:** 10.1186/1472-6831-10-14

**Published:** 2010-06-07

**Authors:** Sasha Scambler, Charlotte Klass, Desmond Wright, Jennifer E Gallagher

**Affiliations:** 1 Department of Oral Health Services Research and Dental Public Health, King's College London Dental Institute, London, UK; 2 Department of Oral Health Services Research and Dental Public Health, Former Senior House Officer, King's College London Dental Institute, London, UK; 3 Consultant in Dental Public Health, National Health Service City & Hackney, Tower Hamlets and Newham, London, UK

## Abstract

**Background:**

The objective of this study was to explore oral health knowledge and beliefs and access to dental care in a culturally distinct Orthodox Jewish community in North London, with a view to informing local health policy.

**Methods:**

A dual method qualitative approach to data collection was adopted in this study utilising semi-structured face to face interviews and focus groups with women from this North London orthodox Jewish community. In total nine interviews and four focus groups were conducted with a purposive sample of thirty three mothers from the community aged 21-58 years. The data were transcribed and analysed using Framework Methodology

**Results:**

Cultural influences, competing pressures and perceptions of hereditary influences, together with a lack of contemporary oral health knowledge are the main factors affecting oral health knowledge and beliefs. This supported an overall perspective of disempowerment or a perceived lack of control over oral health behaviours, both for mothers and their children. Community signposting pointed mothers to dental services, whilst family pressures together with inadequate capacity and capability and generic barriers such as fear and cost acted as barriers. Mothers from this community welcomed community development initiatives from the NHS.

**Conclusions:**

The results of this study provide insight into the challenges of a culturally isolated community who would welcome community support through schools and expanded culturally appropriate opening hours to improve access to dental care.

## Background

### Introduction

The Orthodox Jewish Community in North London is a distinct cultural group held together by a combination of religious observance and cultural practices. It is estimated that the Orthodox Jewish Community in North London comprises around 18,000 members of which 15,800 live in Hackney (accounting for 7.85% of the local population) [1]. The community is growing by 8% per year, has doubled between 1989 and 2008 and is predicted to double again by 2011. The borough of Hackney is ranked as the most deprived local authority in England on measures of income; employment; health deprivation and disability; education, skills and training; barriers to housing and services; crime and disorder and the living environment [2]. High levels of poverty are reported within the Jewish community in Hackney, where 58% below retirement age are receiving a means tested benefit [1]. Socially, however, this group is realtively wealthy in comparison with other ethnic groups in Hackney as any available money is more likely to be used in the provision of community facilities than the accumulation of personal wealth. Large families together with high cost housing results in many families living in cramped conditions.

From the mid 19^th ^century, the north London Orthodox Jewish Community integrated Jews fleeing persecution from Stalinist Russia and Nazi Europe from the mid 19^th ^century. This community is predominantly Hasidic; its congregations represent historical links with particular areas of Eastern Europe in their dress style and worship. Religious observance provides the community's framework and structure for the day and interpretations of religious laws govern all aspects of daily life from food to education and dress to leisure. The community, whilst spread across Hackney sharing a large area with many other communities, is geographically clustered around places of worship and kosher shops and presents as largely homogenous in terms of religious observance, family values and general outlook.

The Jewish Sabbath runs from sundown on Friday to nightfall on Saturday, during which the community refrains from weekday activities such as driving, shopping, using the telephone or visiting the dentist. Members of the community are unlikely to visit a dentist on a Friday, unless in a dental emergency, due to preparations for the Sabbath. The community is also relatively isolated from the wider society excluding use of television, secular media, internet, secular magazines and newspapers. There is access to the wider Jewish community through Jewish magazines and newspapers but very limited access to secular or other religious communities within the wider population. Dental and medical practices accessed tend to be Jewish run, although whether this is through preference, lay referral systems within the community, geography, or a lack of choice is unclear and will be explored further. It is also worth noting that women within this community are less likely than average to have access to or drive a car and so are more likely to access health services within the immediate area or with adequate public transport links.

In general the families within this community are significantly larger than average. The community tends to be segregated by gender; boys and girls attend different schools at both primary and secondary school level. Boys' schools are often private, with curriculums shaped by religious teachings, often in Yiddish, and lengthy school days. Girls' schools, in contrast, whilst still Jewish, are generally state-aided, have a wider ranging secular curriculum and use English. This situation results in the limiting of qualifications available to boys and a resultant limitation in accessible and acceptable occupations. The result is that a large proportion of the community have low incomes. Women tend to maintain greatest contact with the wider community; therefore interaction with the family requires effectively accessing mothers. It is within this context that the results presented below need to be understood.

The Health Improvement and Modernisation Programme 2005-08 [3], identified women and children as priority groups for the reduction of health inequalities. Because no research is published regarding oral health beliefs and practices or dental attendance and access in this Orthodox Jewish community, the findings of this study will facilitate an understanding of these issues. Such knowledge will inform local planners and commissioners of dental services working to enhance access to dental care for this community [4]. This paper, thus, starts with a review of the literature on health and oral health within the Orthodox Jewish community before outlining the methodological approach adopted within the study. The results are then presented alongside an outline of key attributes, beliefs and practices of the community as a whole. The key themes to emerge from the data are presented and explicated contextually allowing for cultural and religious explanations to be incorporated in to, and to illuminate, exhibited beliefs and behaviours. This approach is utilised in the hope that understanding the motivations and beliefs behind oral health practices may support oral health in this culturally distinct community.

### Literature Review

Orthodox Jewish groups are largely unrecognized in the minority health literature in spite of barriers and challenges that they face. There is a very limited pool of literature looking specifically at the impact of Jewish orthodoxy on health/oral health or at the specific needs of minority Jewish orthodox groups in the UK. Studies in Israel [5] and Jerusalem [6,7] have demonstrated a significant relationship between Judaism and health, health behaviour and socioeconomic status. Further, observance of Orthodox Jewish law has been identified as a factor in treatment decision making for some dental patients [8]. The picture is both limited and complex because a number of studies purport to challenge the link between Judaism and poor health/oral health [9-11].

A few studies have also explored health interventions within Jewish, but not orthodox, communities. Torpaz et al. [12] carried out a study of schoolchildren in Israel looking at the efficacy of dental health education programmes. They found that giving children toothbrushes, whether as a stand-alone intervention or as part of a wider education programme, improved standards of oral health. This was a finding echoed in a more recent study [13] which found that the promotion of toothbrushing was an effective means of improving oral health behaviour and oral health of young infants and their parents. This approach was found to be particularly beneficial when accompanied by the distribution of toothpastes and toothbrushes. In addition to school based health education programmes, norms in the Orthodox Jewish community may be challenged by explicitly using its social networks to communicate more positive messages. Henderson's study provides a useful example of how social networks may reinforce or challenge misinformation about health and risk and the complex nature of decision-making about children's health [14].

In England health visitors have reportedly perceived that dental health is neglected among the orthodox Jewish community [15]. Women in the North London orthodox Jewish community often marry at a young age and have large families. The average family in the orthodox Jewish community in Stamford Hill is 5.9 compared to 2.5 in Hackney overall and 2.4 in England and Wales. Such large families have implications for oral health. Local surveys would suggest that whilst oral health in children overall in Hackney is similar to the London average [16], the uptake of dental services is low with approximately 33% accessing dental services within a two-year period [17]. Barriers to dental care are a mixture of beliefs, perceptions, emotions and practicalities, some relating to the individual and some to external factors of service provision. Finch [18] suggests these barriers may include cost, fear, perceived lack of need, travel time, and a feeling vulnerability.

The limited literature on the impact of Judaism on health and oral health and the oral health status of minority Jewish ethnic groups presents a very mixed picture when looking at knowledge, beliefs and behaviour. Perhaps unsurprisingly, socioeconomic status in combination with culture appears to be the key to the relationship between Judaism and health with more deprived communities faring worse than their wealthier counterparts. This relationship is directly relevant to the community participating in this study who are both orthodox Jewish and have a low socioeconomic status.

## Methods

The aim of this study was to explore the importance of oral health and access to dental care in the orthodox Jewish community of North London in support of oral health. A dual method qualitative approach to data collection was adopted and semi-structured face to face interviews and focus groups were conducted with women from the North London Orthodox Jewish Community. In total nine interviews and four focus groups were conducted with purposive sample of 33 women from the community. Women aged 18 years and over who were mothers were invited for inclusion in focus groups or individual interviews. A purposive sampling frame was developed based on dimensions perceived to be relevant a priori such as age, employment status and number of children. Mothers were recruited specifically as in this community mothers are most likely to have access to the wider community through schools etc. Thus mothers are both a key to understanding current beliefs and practices and likely to be one of the key arbitrators of them. In total thirty-three women aged 21-58 years took part in the study. A flexible interview schedule was followed in both interviews and focus groups, most of which were recorded although some participants were unwilling to be recorded.

All data were transcribed and analysed using Framework Method, involving three different stages and processes within an analytical hierarchy [19]. In the first stage, raw data was organised and labelled. 'Descriptive accounts' were then developed to chart the 'range and diversity of each phenonmenon' identified within the data and develop classifications and typologies. The final stage involved the formulation of possible explanations to account for the classifications and typologies identified [19]. Throughout the process the data were checked and rechecked across the levels of abstraction in an iterative process. Data were analysed by Jewish and non-Jewish researchers to ensure a greater understanding of the cultural complexity of the issues raised. Key areas explored included: oral health beliefs; dental attendance behaviour; access issues and recommended changes in support of oral health. The study was based in the community and supported by local religious leaders and City & Hackney Teaching Primary Care Trust. Research Ethics approval was sought and obtained from King's College London Research Ethics Committee [KCL REC 05/06-50].

## Results and Discussion

A total of thirty-three women participated in this study. Of the thirty-one who were willing to report their age, respondents ranged from 21 to 58 years, with an average of 31 and mode of 29 years. At the time of interview 13 women reported that they were in paid employment outside of the home and 19 were not. The number of reported children ranged from one to nine. Three women chose not to respond to that question. Of those who did, 20 participants had more than three children and ten respondents had three or less. With a UK average family size of 2.4 children [20] family sizes within this sample are significantly larger than average [15].

On analysis key themes emerged from the data relating to 'Oral health knowledge and beliefs' and 'Dental access'. It is essential that these themes are understood in the context of this community whose values are significantly different from those of wider society.

### Oral Health Knowledge and Beliefs

The first stage of the interviews and focus groups explored existing oral health knowledge and beliefs, including causes and prevention of oral diseases. Knowledge about oral disease and how to prevent it was very limited. Expressed beliefs did not reflect contemporary knowledge and demonstrated a lack of perceived control over oral health.

#### Lacking contemporary knowledge

When asked about prevention, a minority of participants spoke the importance of brushing teeth at least twice a day, there was very little awareness shown about the importance of the role of diet and sugar consumption and the contribution that fluoride makes to preventing dental caries. The health of primary (deciduous) teeth was not perceived as important as they would be replaced by adult teeth. Furthermore, the need to visit the dentist regularly was mentioned by only a minority of women.

"Front baby teeth just get all rotten. I was trying to be very careful brushing the teeth, she doesn't have bottles to bed, and she doesn't have sugar bottles only milk bottles once a day and not when she's sleeping......they all [dentists] gave me such a what for that I'm not taking care properly". (12 h)

"Those are anyway their first teeth, so even if they get decay it's not the end of the world sort of thing, it doesn't go down to the roots or anything." (14 h)

These quotes show a strong lack of understanding of the value of oral health in infants and the benefits of positive oral health related behaviours for future oral health.

When asked about the causes of poor oral health, a link with pregnancy was reported by some participants.

"I find it with every child I have problems with one of my teeth". (18 h)

"It's like my first outing to the, after having a baby is to the dentist." (34 h)

#### Hereditary influences

Part of the perceived lack of controlwas demonstrated by the view that tooth decay was a hereditary process rather than a shared cultural/behavioural issue, and it was suggested that poor teeth in parents or grandparents could be seen in children.

"I've got half the family with excellent teeth, then half the children always have to visit the dentist regularly. We can't help it." (15 h)

#### Culturally influenced diet

The role of diet within the culture was generally deemed more important than the risks to health or oral health. There was a general lack of awareness, alongside a reluctance to admit, the full impact of diet on oral disease. Sweets were used within the community as a reward, and sweets, cakes and sugar laden foods were seen as a staple and important part of religious ceremonies and celebrations. The pervasiveness of high sugar foods within the community was presented as the reason for not, or being unable to limit sugar consumption. Sugar consumption, and sweets in particular, were highlighted as an issue and some participants spoke about the inability to prevent their children from obtaining sweets which are readily available in kosher shops.

"You know a lot of kosher shops, and stuff like that, have mounds of sugary foods. I hate going shopping with the children; they just think they have to put everything into the basket. It's so tempting for them, and it's in your face." (15 r)

Both the lack of general knowledge and awareness of positive and negative oral health behaviours and the particular attitude towards diet need to be understood in the context of a media isolated community. Health promotion campaigns conducted through any form of secular media are unlikely to reach this community. In addition, in a largely homogenous and segregated community, shaped by religious observance where food is integral to ceremonies and celebrations, messages about diet and sugar consumption need to be handled sensitively. Furthermore, messages need to be made accessible to the community through targeted information and campaigns in appropriate media such as Jewish papers, or through community groups and schools. The availability of sugar in the wider environment is a challenge for society as a whole [21], as sweets have also been shown to be promoted in both large and small community shops, and has been the subject of a number of high profile public health campaigns [22]. In addition, the consumption of sweets and high sugar food is an international problem and not just a national on [23]. In this study there was talk about school health eating policies being useful within girls' schools but little evidence of direct action being taken to reduce sugar consumption by the community as a whole.

#### Competing priorities

In common with the lack of knowledge and related beliefs, competing priorities led to the widespread 'non-prioritisation' of oral healthcare within the home. Prioritising oral healthcare relates to the emphasis placed on oral hygiene and oral healthcare within the family. In this sample this was encapsulated in the supervision of toothbrushing. In common with the lack of knowledge and related beliefs, competing priorities led to the widespread non-prioritisation of oral healthcare within the home to the extent that mothers took an active role. A minority of mothers talked about the importance of supervising their children's toothbrushing and oral hygiene practices but many spoke of a lack of time to supervise their children's oral hygiene.

"There is something with larger families, you know it is hard to stay on top of everything and teeth become secondary. A lot of mothers know what to do, they just can't get there." (17 t)

The common solution to this problem was to delegate supervision to older children. Otherwise activity went unsupervised. In summary, this group of mothers demonstrated a lack of contemporary knowledge, cultural influences on diet, problems with competing priorities, and a perceived lack of control over oral health. They welcomed community support.

### Access to dental care

Mothers reported that they and their families did not regularly attend a dentist but overall demonstrated a sense of 'doing what they can'. Only a minority suggested that they attended a dentist at the traditional interval of 'every six months'. A number of issues were raised relating to the provision of dental care as well as problems with accessing dental services related to family pressures, together with generic barriers to care.

#### Community signposting

Within the community a dentist was usually chosen through personal or family recommendation and a limited number of dentists working within the area were deemed acceptable.

"You just go with them because its kind a community thing. You hear she goes, she goes and you all just go back. I go because my friend goes and she goes and he goes, not that I know if, I don't even trust them." (13 r)

"I just moved here and found him through word of mouth. You go where your family goes. We do not want to go to someone we do not trust. You can hear a name at the Jewish centre and then everyone goes." (25 m)

It is worth noting here that the word of mouth is the most common means of finding dental services amongst adults [24], and is not unique to this community.

No preference was stated when participants were asked about the importance of ethnic background in relation to practitioner choice, but a number of participants stated that they would prefer a male dentist. Men were generally felt to be more capable and decisive and anecdotal evidence was provided to support this assertion.

"I prefer a male, they are more sure of themselves...a man knows his stuff - it's different." (28 r)

"She's a lady dentist...she's scared to do it." (13 n)

It is worth noting here that whilst participants stated no preference for ethnically specific practitioners the vast majority attended Jewish dentists or dental practices. Once again this ties in with the fact that referrals are passed through the community and also to the lack of contact with many external sources of information such as the list of NHS dentists which is available on the internet through NHS Choices [25].

#### Inadequate capacity and capability

Both the capacity and capability of local dental care were raised as issues by regular and symptomatic attenders alike, focussing on long waiting times, both for appointments and at the surgery itself:

"Why I don't go with my children...when the waiting time is three hours I don't have three hours to spare. That is the only reason." (18 k)

"Most people wait until it is an emergency. I have to wait ages for an appointment. It is difficult to take the children along as well". (27 o)

Dentists accessed by this community were reportedly overstretched due to their limited numbers and face a challenging patient population in an area of London where space is at a premium. Concerns covered surgeries with a lack of play areas and toys to serve this community.

"open up children centres which are friendly to children, toys (so that) children excited to go and not scared." (4 s)

Organisational issues were also mentioned, covering a lack of Sunday clinics and flexible opening times and direct and indirect costs of treatment. The lack of access to salaried community dental services was also raised. Whilst many of these issues are relevant amongst the wider population, lack of play areas and toys, long waiting times and costs are particularly relevant when considering a community where the majority of whom have large families.

#### Generic barriers to care

In addition to availability, other barriers identified included perceived cost of treatment; travel costs; waiting times, poor dentist patient communications skills [18] and the pressures resulting from having large families. However, it is possible that the lack of knowledge about dental charges has exacerbated a 'fear of cost' as all children and a number of adult members could be eligible for free dental treatment. Again the experiences raised are pertinent when considered in the context of the community in which these families live. These factors are further exacerbated by issues raised by participants about the organisation and provision of dental care and highlight the impact of multiple barriers to care. Respondents identified the need for extended hours and Sunday opening, together with reducing direct and indirect costs of treatment. Indirect costs could be reduced by provision of local services requiring less travel and greater capacity which would reduce the pressure on services and thus waiting times.

### Welcoming community support

One of the final themes to emerge from the data concerned both the ways in which people behave in relation to their oral health and the development of oral health knowledge. Respondents highlighted a lack of time when asked about teaching their children about oral healthcare and it was widely felt that the schools should play a significant role in teaching these kinds of health related topics.

"The children have a lack of knowledge. They should be coming in to schools. Then they would come home from school saying I want to brush my teeth." (24 t)

It was suggested that school health programmes could form an important community initiative to develop personal skills of children who would be better able to care for their own health and possibly strengthen family knowledge about oral health.

"Dentists should come round the schools to help them to brush their teeth, to show the children ho to brush their teeth and to encourage them, because I think the schools do, the children get a lot of encouragement through the teachers definitely." (13 s)

This was evidence of the willingness of the community to receive external support. This could be perceived as being at odds with both the central role of family within this community and its role in teaching and nurturing children. Alternatively it could be seen as an empowering solution in support of the community, in recognition of the respondents' desire to enhance the priority of oral health within the population. Thus the context is germane to making sense of the behaviour exhibited and to determining ways of overcoming these boundaries.

### Limitations and Strengths of the Study

Whilst it is acknowledged that this study is limited by its sample size and the fact that fieldnotes replaced recorded transcripts where permission was not granted for recording, the findings represent a unique and valuable insight into this particular community. This study is an important contribution to the literature on the oral health beliefs, behaviours and needs of the orthodox Jewish community in North London. It further adds to understanding of other culturally isolated communities in socially deprived areas. As an indepth case study of an isolated community which appears difficult to access, the study provides a unique insight into the specific needs of women and families from this community. The direct link with the City & Hackney Teaching Primary Care Trust has allowed for the development of culturally acceptable and appropriate interventions in partnership with the community. The findings provided the necessary information to enable the local state-funded health organisation to develop services that meet the needs of this population. To this end it has commissioned a needs assessment of health in this community the dental aspects of which will be informed by this study.

## Conclusions

The data presented in this paper can be seen to present a picture of a distinct and socially isolated cultural community which holds a way of life focused on maintenance of tradition, exhibiting, plausible but sometimes inaccurate health-related beliefs. Interestingly, however, despite feeling little autonomy to help themselves, support from outside the community is welcomed in relation to oral health. By contextualising the data we can build a useful picture of a community tied in to a way of life whose chosen distance from modern media may, perhaps inadvertently, perpetuate limited knowledge of health related matters. National and International evidence suggests that parents perceptions of efficacy in controlling the sugar snacking habits and oral hygiene behaviour of their children have been shown to be significant [26]. Furthermore, parental perceptions of efficacy are significant predictors of the development of favourable behaviours in children [26].

Bearing this in mind, we can look at ways of positively and practically intervening to ensure that health information is made available to members of the community and to facilitate access to and use of dental services. Recommendations from this study include the development of a culturally sensitive oral health promotion programme, targeted at children and mothers, to improve oral health knowledge and support behaviour change and a review of capacity and capability availability of dental services for this community to include both routine and emergency dental services and extended hours and Sunday opening.

The findings and recommendations of this study are not intrinsically counter-cultural as Judaism obliges its followers to protect themselves and others from illness and disease. Stewart-Freedman & Kovalsky [27] go so far as to say that "The Oral Law specifically states that 'All of Israel is responsible one for the other'" [Shavuot 39a; Sotah 37a; Rosh Hashanah 29a]. They go on to suggest that the hierarchical and disciplined structure of most Hassidic Jewish orthodox groups make them ideally placed to promote preventative health and oral health measures and that "more needs to be done in future to understand the social structure and establish lines of communication with the key contact points of this and other hard-to-reach communities" [[27]: p2]. Whilst further research is needed, particularly looking at the beliefs and attitudes of men as well as women within the community, this study and the work that has been done developing links and establishing school based oral health education programmes represents a very important step in developing understanding of a community and making a real difference to the oral health behaviour and oral health of a minority cultural group.

## Competing interests

The authors declare that they have no competing interests.

## Authors' contributions

CK was involved in the design of the study, fieldwork, data analysis and contributed to the write up of the paper. DW was involved in the design of the study and contributed to the write up of the paper. JG was involved in the design of the study, data analysis and contributed to the write up of the paper. SS was involved in the analysis and write up of the paper. All authors read and approved the final manuscript.

## Pre-publication history

The pre-publication history for this paper can be accessed here:

http://www.biomedcentral.com/1472-6831/10/14/prepub
